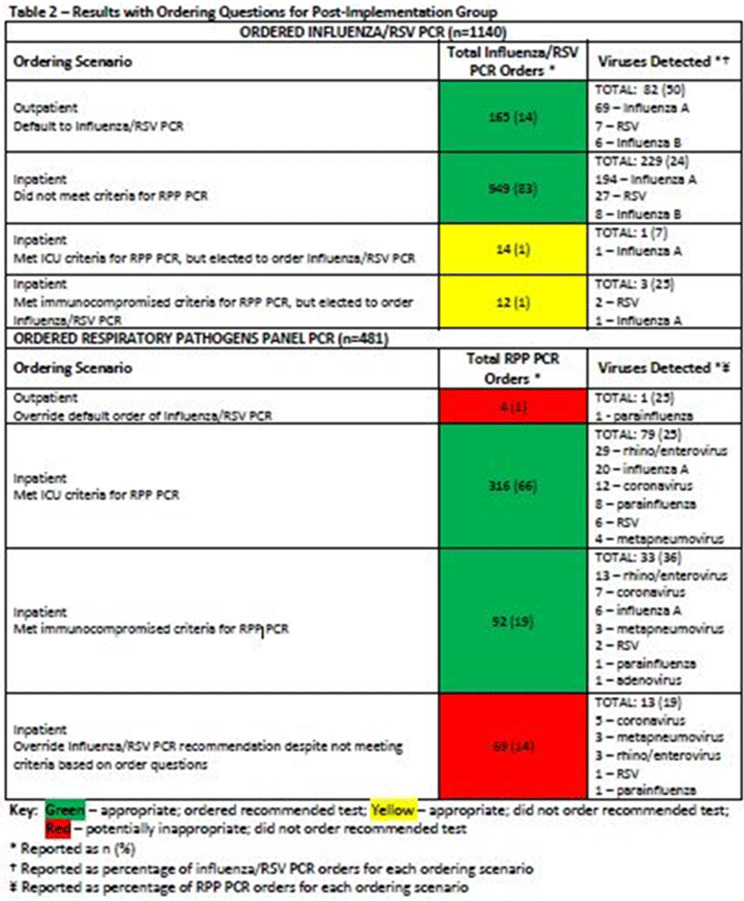# Diagnostic Stewardship of Respiratory Pathogen Panel Utilization

**DOI:** 10.1017/ash.2021.114

**Published:** 2021-07-29

**Authors:** Amy Kressel, Megan Cheatham, Amy Chang

## Abstract

**Background:** Diagnostic stewardship modifies the ordering, performing, and reporting of diagnostic tests to optimize clinical care and infection prevention while conserving healthcare resources. Timely and accurate diagnosis of respiratory virus infections can optimize the use of antibiotics, antivirals, ancillary tests, and inpatient beds. Influenza-like illnesses (ILIs) are frequently caused by viruses. However, before COVID-19, specific antiviral medication was commonly used only for the treatment of influenza virus infections. **Methods:** Eskenazi Health (EH) had 2 respiratory PCR assays: influenza/RSV ($58.18 per assay) and a 20-pathogen respiratory pathogens panel (RPP) ($129 per assay). An inpatient ILI algorithm was developed and implemented in the electronic health record (EHR) in October 2018 to guide the selection of the appropriate assay (Figure 1). Ambulatory testing defaulted to the influenza/RSV assay. Prescribers retained the ability to override recommendations. We performed a retrospective chart review of all orders for RPP and influenza/RSV assays before implementation of the ILI algorithm (October 1, 2017, to September 30, 2018) and after implementation (October 1, 2018, to September 30, 2019). The primary end point was the number of RPP assays ordered. The secondary end point was the appropriateness of RPP assays ordered (ie, met ≥1 criteria) and number of influenza/RSVs assays ordered with virus detected. **Results:** Before the implementation of the intervention, 1,882 orders were reviewed. After implementation 1,621 orders were reviewed. All influenza/RSV and RPP assays were included if they were ordered between October 1, 2017, and September 30, 2019, at EH. There were no exclusion criteria. After implementation, RPP assays decreased ~40% (Table 1), with associated cost savings of $35,368.68 (22.6% of total assay costs; $163,742.88 before implementation and $128,374.20 after implementation). Although some of this reduction could be attributed to the lower number of overall assays ordered, the 40% reduction in RPP assays exceeded the 14% decrease in overall orders, demonstrating improvement in utilization of RPP assays. A corresponding increase in influenza/RSV assay orders was not observed; both groups had similar total influenza/RSV orders. Both groups also had similar percentages of viruses detected with influenza/RSV and RPP (33% before vs 31% after). After implementation, 1,522 (94%) of 1,621 orders followed the recommendations of the ILI algorithm (Table 2). Several prescribers ordered influenza/RSV despite the patient meeting criteria for RPP assay; of these 26 assays, 4 (15%) resulted in virus detection. Of the 73 instances in which prescribers bypassed recommendations for the influenza/RSV assay and ordered an RPP assay, 14 (19%) of the assays resulted in virus detection; only 1 of 14 was a virus that would have been detected by the influenza/RSV. We were unable to identify any trends that would assist in developing additional order questions to capture these patients. **Conclusions:** Implementation of the ILI algorithm was associated with high adherence, improvement in the appropriateness of ordering, and significant cost savings.

**Funding:** No

**Disclosures:** None

**Figure 1.**
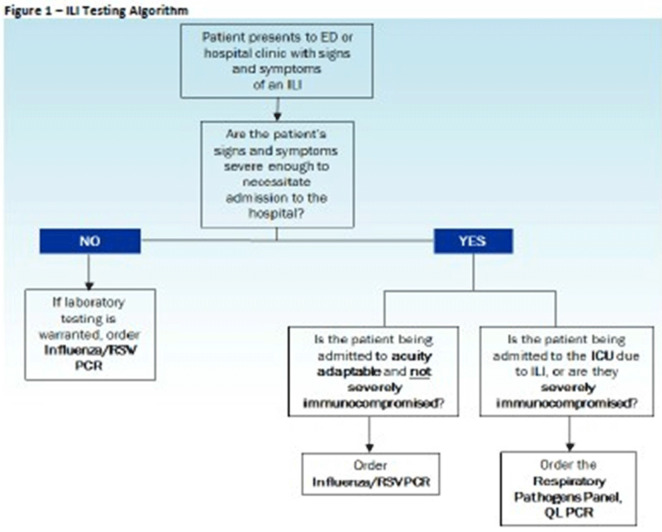

**Table 1.**
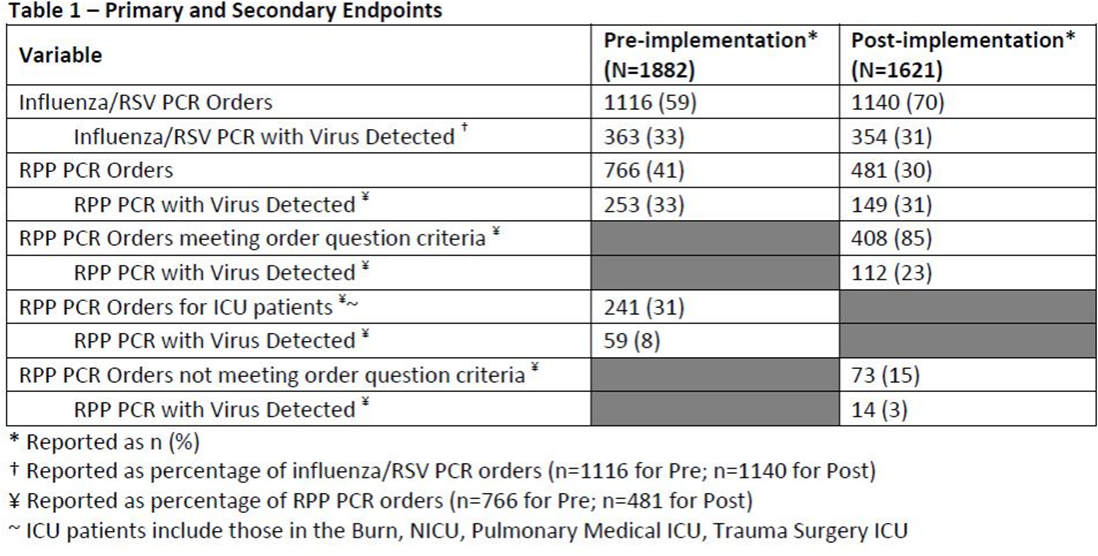

**Table 2.**